# Neuroendocrine carcinoma causing common bile duct obstruction: a case report

**DOI:** 10.1093/omcr/omaf011

**Published:** 2025-03-28

**Authors:** Richard Shamoon, Osman Elhassan, Lujain Al-Emadi, Abdulwahab Zabara, Mahir A Petkar, Sarah Sayed, Osama H Mohammad

**Affiliations:** Department of Internal Medicine, Hazm Mebaireek General Hospital, Street 33, Industrial Area, Al-Rayyan, Doha, Qatar; Department of Medical Education, Hamad Medical Corporation (HMC), C-Ring Road, Hamad Medical City, Doha, Qatar; Weill Cornell Medicine, Al-Luqta St, Education City, Al-Rayyan, Doha, Qatar; Department of Diagnostic Radiology, Hazm Mebaireek General Hospital, Street 33, Industrial Area, Al-Rayyan, Doha, Qatar; Department of Laboratory Medicine and Pathology, Hamad Medical Corporation (HMC), C-Ring Road, Hamad Medical City, Doha, Qatar; Department of Laboratory Medicine and Pathology, Hamad Medical Corporation (HMC), C-Ring Road, Hamad Medical City, Doha, Qatar; Department of Internal Medicine, Hazm Mebaireek General Hospital, Street 33, Industrial Area, Al-Rayyan, Doha, Qatar

**Keywords:** periampullary neoplasms, common bile duct obstruction, periampullary carcinoma, jaundice

## Abstract

A 46-year-old male with no comorbidities was referred to our hospital because of jaundice and elevated LFT markers. After further investigations, he underwent magnetic resonance cholangiopancreatography (MRCP), which revealed a hypo-enhancing periampullary mass measuring 15 mm in size causing common bile duct (CBD) dilatation of 12 mm in cross diameter with intrahepatic biliary obstruction, which explained the patient’s symptoms. Side-view endoscopy was performed to obtain a specimen of the mass. Further histopathological workup revealed poorly differentiated neuroendocrine carcinoma (NEC). A multidisciplinary team (MDT) was conducted, and the patient was planned to undergo positron emission tomography-computed tomography (PET-CT) scan to investigate any further organ metastasis. Unfortunately, the patient missed his upcoming appointments and was lost to follow-up. Nevertheless, more research is needed to understand pathogenesis and the best course of management for small periampullary NETs.

## Introduction

Neuroendocrine carcinomas (NECs) represent a heterogeneous group of malignancies originating from neuroendocrine cells, dispersed throughout the body. These cells possess the characteristics of both nerve cells and hormone-producing endocrine cells, enabling them to release hormones in response to neural signals [1, 2]. NECs are malignant neuroendocrine neoplasms (NENs) with poor differentiation and high proliferation rates (Ki-67  >  20% and/or mitotic count > 20 per 10 high-power fields). Accordingly, NECs show an increased tendency for distant metastasis, leading to poor prognosis [2]. Although neuroendocrine tumors (NETs) are commonly identified in the gastrointestinal tract and lungs, their occurrence in the biliary tract is particularly rare and poses significant diagnostic and therapeutic challenges [3].

We present the case of a 46-year-old male diagnosed with NEC causing CBD obstruction. This case emphasizes the importance of a multidisciplinary approach and contributes to the limited literature on this rare entity, providing insights for clinicians to manage similar cases.

## Case report

A 46-year-old Indian male presented to the emergency department with jaundice for one day and generalized body ache for two weeks. The patient had no gastrointestinal or urinary symptoms. The patient also reported a weight loss of 5 kg in the previous 6 months.

**Figure 1:**
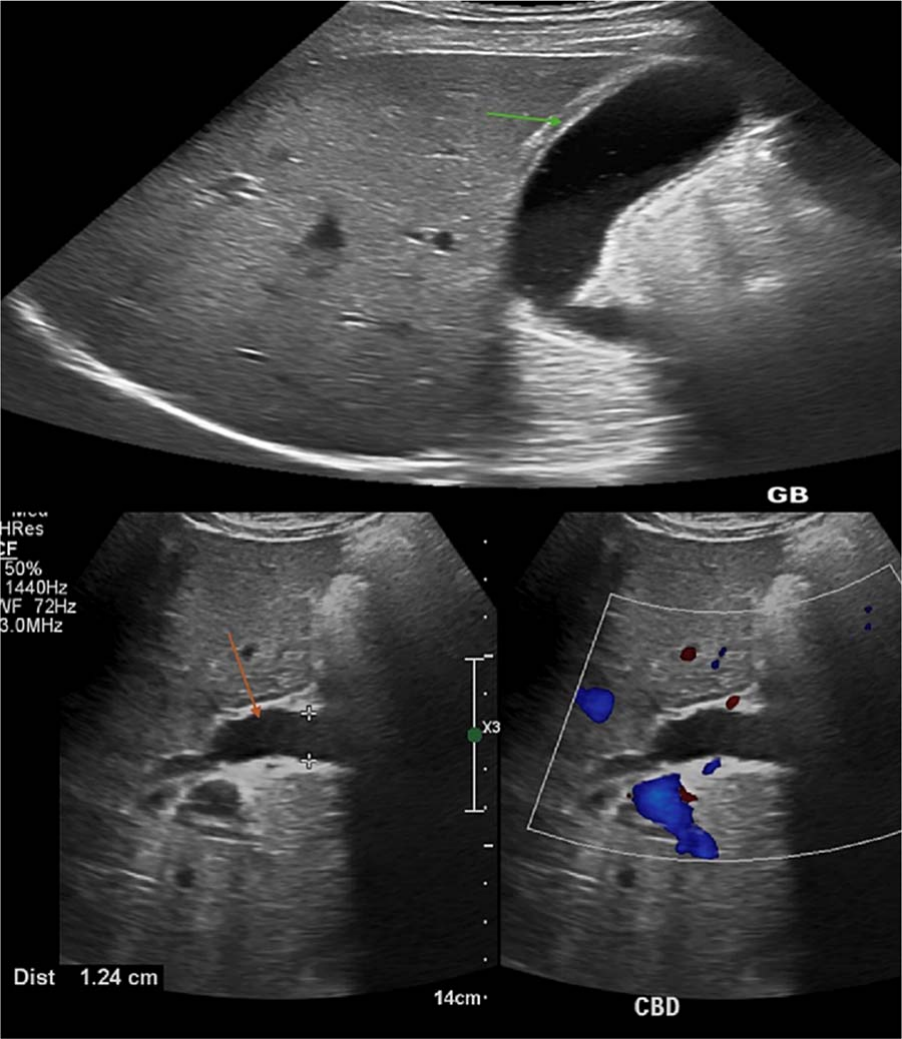
Abdominal axial ultrasound of the liver and gallbladder shows thick edematous gallbladder wall and a dilated common bile duct with intrahepatic biliary dilatation.

On presentation, the patient was oriented, stable, and comfortable. On physical examination, the patient had obvious jaundice, but no abdominal tenderness or hepatosplenomegaly. An ultrasonography revealed significant dilatation of the common bile duct (orange arrow) measuring 12.4 mm, without obvious ductal stones. The gallbladder also showed distension and edematous wall (green arrow) resembling features of cholecystitis, but no sizable stones could be detected. (Fig. 1).

Laboratory workup revealed hyperbilirubinemia and elevated hepatic and pancreatic enzymes (Table 1), while his hepatitis serology markers (Hep A IgM, Hep B Core Ab, Hep B Surface Ab, Hep B Surface Ag, Hep C Ab, Hep E IgM) were all negative.

**Table 1:** Laboratory workup results after admission.

Parameters	References Value
White blood cells	7.4 × 10^3/ul
Hemoglobin	13.1 gm/dl
Total Bilirubin	92 umol/l
Direct Bilirubin	82 umol/l
Total Protein	85 gm/l
Alkaline Phosphatase	659 U/l
ALT	457 U/l
AST	220 U/l
Lipase	543 U/l
HbA1C	6.0%

**Figure 2:**
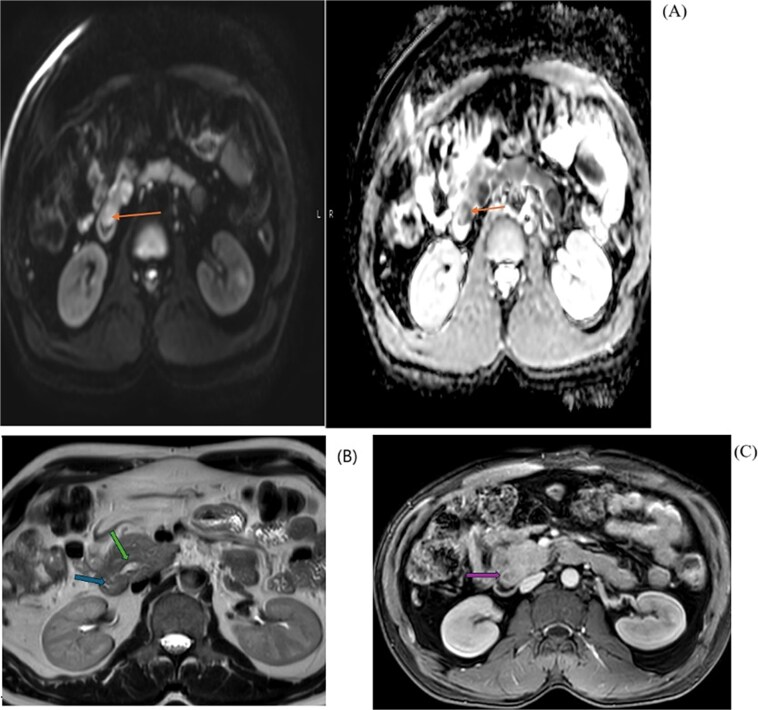
MRI diffusion/ADC map (A) shows restriction pattern of the periampullary lesion reflecting bright signal on diffusion and dark signal on ADC map. MRI axial T2 haste image at the level periampullary region (B) shows rounded isointense lesion. And post IV contrast axial MRI image (C) shows hypo enhancement of the peri-ampullary mass.

Based on the above investigation, a MRCP was planned. An axial view (Fig. 2) showed a hypo-enhancing periampullary mass lesion, which was seen showing restricted diffusion (orange arrow) measuring approximately 15 mm in size causing abrupt cut-off of the distal CBD with post IV contrast injection shows hypo enhancement of the periampullary mass (purple arrow). MRI Axial T2 Haste image at the level periampullary region shows a rounded isointense lesion in the periampullary region bulging into the lumen of the 2^nd^ part of the duodenum (blue arrow), with dilated pancreatic duct (green arrow).

**Figure 3:**
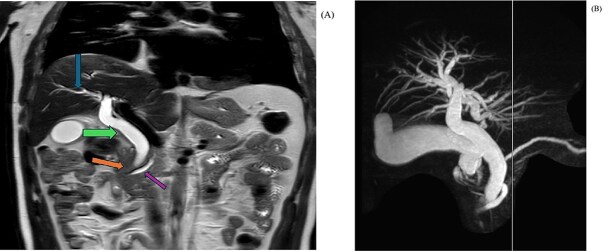
MRI coronal T2 haste (A) showing abrupt cut-off of the distal CBD with a dilated common bile duct, mild degree of intrahepatic biliary dilatation and mild dilatation of the pancreatic duct. MIP image (B) shows the cut off at the distal CBD, with severe dilatation of the extrahepatic and intrahepatic biliary tree, as well as dilated pancreatic duct.

In coronal view at the same level (Fig. 3), a MRI T2 Haste shows abrupt cut-off of the distal CBD (orange arrow) with a dilated common bile duct (green arrow) and a mild degree of intrahepatic biliary dilatation (blue arrow). The CBD proximal to the level of obstruction measures approximately 12 mm in caliber. Mild dilatation of the pancreatic duct is noted giving the double duct sign (purple arrow) with the main pancreatic duct measuring approximately 6.7 mm in the region of the pancreatic head.

MIP image (B) from MRCP confirms the cut off at the distal CBD, with severe dilatation of the extrahepatic and intrahepatic biliary tree, as well as dilated pancreatic duct.

**Figure 4:**
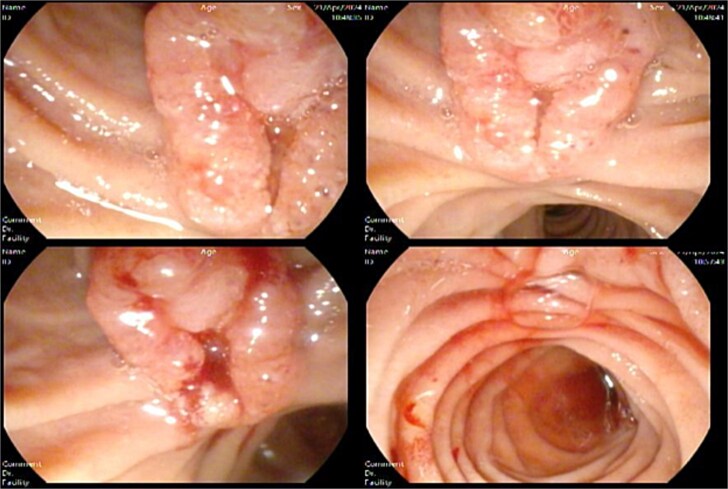
Side view endoscopy showed an ulcerated ampullary mass.

Suspecting malignancy, a side view endoscopy was planned, and tumor markers (AFP, CA 19-9 and CEA) were ordered, which showed an ulcerated ampullary mass of ~1.5 cm in size causing common bile duct obstruction (Fig. 4) and a normal stomach and esophagus.

Tumor markers panel revealed elevated CA 19-9 levels (table 2).

**Table 2:** AFP, CA 19-9 and CEA tumor markers results.

Tumor Marker	Results
AFP	3 IU/ml
CA 19-9	87.2 U/ml
CEA	3.0 ug/l

**Figure 5:**
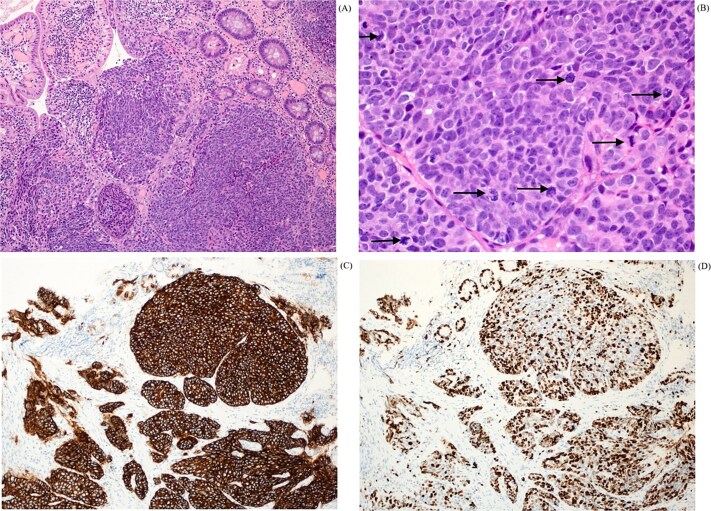
Histopathological findings in H&E x 4 (A) shows variable sized nests of tumor in the lamina propria. H&E x 40 with mitosis (B) shows nuclear pleomorphism with frequent mitotic activity (black arrows). The tumor cells are strongly and diffusely positive for synaptophysin (C) and Ki-67 immunostain (D) displaying high proliferative index of more than 70%.

A biopsy was taken and sent to the histopathology department. Histology from the periampullary mass biopsy (Fig. 5) revealed variably formed nests of tumor in the lamina propria, composed of large pleomorphic tumor cells, exhibiting granular nuclear chromatin (A). Mitosis was frequent and easily identifiable (B). The tumor cells were positive for synaptophysin (C) and chromogranin, and exhibited high proliferative index, with Ki-67 more than 70% (D). The morphology, immunohistochemistry and high proliferative index were in keeping with a diagnosis of poorly differentiated neuroendocrine carcinoma.

An MDT was conducted for the patient, and a PET-CT was ordered to plan further management. Unfortunately, the patient missed his appointment, and additional attempts to contact him failed.

## Discussion

According to the 2022 WHO classification of tumors [4], neuroendocrine neoplasms (NEN) [formerly known as neuroendocrine tumors (NETs) before reclassifying them in the 2019 WHO classification of tumors [5]] include well-differentiated NETs and poorly differentiated neuroendocrine carcinomas (NECs). NECs are poorly differentiated high-grade epithelial neoplasms, which differ from periampullary neuroendocrine tumors (NETs) by their molecular mutations, as NECs predominantly have TP53 or RB1 mutations, while MEN1, DAXX and ATRX are the entity-defining mutations for well-differentiated NETs [5].

Periampullary NECs are extremely rare tumors which represent a small part of the gastrointestinal NECs (0.9%–2% of primary ampullary tumors) and most of the time, they are localized in the ampullary region [6]. To be defined as a tumor of the ampulla of Vader, it should arise in the last centimeter of the common bile duct [7]. Even though they are relatively rare, they often tend to be more aggressive than duodenal NENs [8, 9]. While our patient was a middle-aged man, most cases occur later in life (age 50s and 60s) and tend to be more predominant with women than men (3:1 predominance) [10].

Clinically, periampullary NENs can have the same presentation of symptoms of extrahepatic cholangiocarcinoma and pancreatic adenocarcinoma, which is most commonly is jaundice, abdominal pain and malaise, but other symptoms like steatorrhea, weight loss, or melena can also be present [11]. The Modalities for diagnosis can be endoscopic ultrasound (EUS), endoscopic retrograde cholangiopancreatography (ERCP), fine-needle aspiration cytology (FNAC), and computed tomography (CT) can be used for staging [12]. In a retrospective study of 20 patients, it was found in half of the cases, the tumors did metastasize. Paradoxically, the smaller the tumor, the higher the chance for it to metastasize (66% for tumor < 1 cm, 50% for tumor 1–2 cm and 46% for tumor > 2 cm) [13].

As for treatment options, there is no consensus on the best course of management, which is expected given the small number of cases reported. However, most doctors recommend surgical resection, like the European Neuroendocrine Tumor Society, which believes it’s the most reliable curative treatment [14]. Two options for surgical resections are local resection and pancreaticoduodenectomy (PD). In a study comparing both, local resection was performed generally in tumors less than 2 cm and PD in larger ones. 3 of the 52 patients who underwent PD died of postoperative complications. Compared to 21 of the 22 patients who remained alive without tumor recurrences after local resection [15].

In alternative treatments, endoscopic papillectomy (EP) had shown promising results for small tumors without metastasis (less than 10 mm) [16]. While in metastatic or inoperable cases, systemic chemotherapy is indicated. Remission was induced in 55%–80% of patients with cisplatin/carboplatin and etoposide co-therapy [17].

For the prognosis, it was found that the 10-year survival rate is 71% for the well-differentiated tumor. However, it is 15% for the poorly differentiated NECs [3].

In conclusion, the case reports for periampullary NECs and NETs are relatively few and limited in information, and more research is needed to devise the best evidence-based management.

## Consent

Written consent was taken for the publication of this case report.

## Guarantor

Dr. Osama H. Mohammad.

Dr. Richard Shamoon.
